# Cytogenotoxicity biomarkers in fat snook *Centropomus parallelus* from Cananéia and São Vicente estuaries, SP, Brazil

**DOI:** 10.1590/S1415-47572009005000007

**Published:** 2009-01-17

**Authors:** Aline A. Kirschbaum, Robson Seriani, Camilo D. S. Pereira, Andrea Assunção, Denis M. de Souza Abessa, Matheus M. Rotundo, Maria J. T. Ranzani-Paiva

**Affiliations:** 1 Laboratório de Ecotoxicologia, Universidade Santa Cecília, Santos, SPBrazil; 2 Laboratório de Ecotoxicologia Marinha, Instituto Oceanográfico, Universidade de São Paulo, São Paulo, SPBrazil; 3 Instituto de Pesca, São Paulo, SPBrazil; 4 Centro Universitário São Camilo, São Paulo, SPBrazil; 5 Núcleo de Estudos em Poluição e Ecotoxicologia Aquática, Universidade Estadual Paulista, São Vicente, SPBrazil; 6 Laboratório de Pesquisas Biológicas, Universidade Santa Cecília, Santos, SPBrazil

**Keywords:** cytogenotoxicity, nuclear abnormalities, micronuclei, *Centropomus parallelus*

## Abstract

The aquatic environment receives many contaminants that can induce damages at the molecular, biochemical, cellular and physiological levels. *Centropomus parallelus*, an important food resource for local populations, is a predator fish that feeds on small fishes and benthic invertebrates, thus being vulnerable to the bioconcentration and biomagnification processes. This study aimed to evaluate cytogenotoxic responses in erythrocytes from *C. parallelus* juveniles collected in the Cananéia and São Vicente estuaries, both in winter and in summer. After anesthesia, blood samples were collected by caudal puncture. Blood smears were prepared on glass slides and stained with May-Grünwald-Giemsa dye. Two thousand cells were analyzed per slide (1000x), and nuclear abnormalities (NA) and micronuclei (MN) were scored. The São Vicente sample showed MN and NA frequencies (%/1000 cells) of 0.325 and 3.575, in winter, and of 0.125 and 2.935 in summer respectively; the Cananéia sample showed frequencies of 0.0325 and 0.03, in winter, and of 0.065 and 0.355 in summer, respectively. The rates found in São Vicente were significantly higher than those found in Cananéia, evidencing that the levels of pollution in that estuary were high enough to induce genetic damages.

The levels of aquatic pollution are increasing along the Brazilian sea coast, mainly due to the discharge of industrial and domestic effluents and to the presence of other human activities, such as ports, landfills and mining, among others (Sousa *et al.*, 2007).

The São Vicente estuary is a part of the Santos Estuarine System (SES) that presents multiple sources of contamination, such as industries, sewage outfalls, stormwater drainage, the port of Santos, as well as illegal landfills and dumping sites. Recent researches in the São Vicente estuary have shown that many contaminants are present there at concentrations high enough to cause biological effects (Cesar *et al.*, 2006; Abessa *et al.*, 2008; and 2001 water quality report for the São Vicente estuary).

According to the water quality reports for the São Paulo Costal region for 2005 and 2006 (see Internet Resources), in sediment samples derived from the Santos and São Vicente Estuarine System, some of those substances, such as copper (Cu), zinc (Zn) and lead (Pb), presented levels between the threshold effect level (TEL) and the probable effect level (PEL) (limits established by the Canadian Council of Ministers of the Environment, 1999), where the highest values were 53.8, 125 and 105 μg.g^-1^, respectively. The levels of mercury (Hg) found were above PEL (1.36 μg.g^-1^). Also, polyaromatic hydrocarbons (PAHs), such as benzo(a)pyrene and dibenzo(a)anthracene, were present at levels above TEL, showing values of 198-206 and 87.4-90.9 μg.kg^-1^, respectively. These contaminants may induce damages at the molecular, biochemical, cellular and physiological levels, leading to reproductive impairment, abnormal development, cancer, lethal mutations, as well as to an increase or decrease of the genetic variability (Dickmann *et al.*, 2004). In contrast, Cananéia is an unpolluted estuarine area (water quality reports for the São Paulo Costal region for 2005 and 2006), located in a legally protected area, that has been used as a reference site.

In marine and freshwater ecosystems, cytogenotoxicity assays such as those evaluating micronuclei and nuclear abnormality rates have been widely employed to biomonitor wild areas with different levels of contamination, using a variety of organisms as marker species, ranging from mussels to fishes (Rodriguez-Cea *et al.*, 2003). Micronuclei are the result of genotoxic damage, evidenced through small masses of chromatin loose in the erythrocyte cytoplasm during mitosis, whereas nuclear abnormalities caused by cytotoxic damage are evidenced by the abnormal nuclear shape of the erythrocytes that can be characterized as blebbed, lobed, notched, vacuolated or conical, as described by Carrasco *et al.* (1990).

The purpose of this study was to evaluate cytogenotoxic responses in erythrocytes of *Centropomus parallelus* juveniles from the São Vicente and Cananéia estuaries (Figure 1) by analyzing the incidence of micronuclei (MN) and nuclear abnormalities (NA) (Figures 2.A and B), in order to assess chromosome damages. The results obtained for winter and summer between and within the sites were compared, aiming to determine seasonal and local differences.

The fat snook (*Centropomus parallelus)* inhabits shallow coastal waters and estuaries from the south of Florida to the Brazilian coast (tropical and subtropical waters). This specie does not accomplish large displacements, and its juveniles can reach the rivers and remain for some periods in freshwater (FAO Fisheries Department,1978). Besides small fishes, this species also feeds on benthic organisms, which makes it vulnerable to the bioconcentration and biomagnification of contaminants. *C. parallelus* was selected for this study because it represents an important food resource for the local coastal population, and the good quality of its meat makes it also become a valuable economic resource: one kilogram reaches about 10 US dollars in the local market. The nominal catches in Brazil have increased every year since 1990, reaching a peak of 5000 tons in 2000, according to the FAO Catches List (*FishBase* 2007).

To evaluate the cytogenotoxic responses, ten juvenile fishes each were collected from the Cananéia estuary in winter 2004 (July) and summer 2005 (January); similarly, ten animals from the São Vicente estuary were collected in winter 2006 and another ten in summer 2007. The fishes were transported to the laboratory and acclimatized during 30 min. After anesthesia with clove oil (Delbon, M.C. Ação da benzocaína e do óleo de cravo sobre os parâmetros fisiológicos de tilápia, *Oreochromis niloticus.* Master thesis. Centro de Aqüicultura da UNESP – Universidade Estadual Paulista Júlio de Mesquita Filho, 2006), blood samples were collected by caudal puncture with previously heparinized needles and syringes. Blood smears were prepared on glass slides and stained with May-Grünwald-Giemsa dye (Rosenfeld, 1947), following the procedure described by Ayllon and Garcia-Vazquez (2000, 2001) and Rodriguez-Cea *et al.* (2003). Two thousand cells were analyzed per slide per animal by optical microscopy (1000x). Mononuclear erythrocytes were analyzed to evaluate the rates of MN and NA. The NA score was based on the classification presented by Carrasco *et al.* (1990).

Biometric parameters (total length and weight) were analyzed before the blood collection. Sex determination and observation of gonadal maturation were made after the blood collection.

The Shapiro-Wilk Test for normality was performed. The results obtained for MN and NA were compared by using Student's *t*-test to detect differences at the 0.05 level of significance, between and within sites and in the different seasons. The influence of seasonality, biometric parameters, sex and gonadal maturation on the biomarker rates was determined using the same test.

The data regarding biometric parameters, sex and gonadal maturation stages are presented in Table 1.The frequencies of the two biomarkers for both areas and seasons studied are presented in Table 2. Data normality was observed. The rates obtained for both MN and NA in fishes from São Vicente were higher than those observed among the fishes from Cananéia. These frequencies were 10X higher for the MN and 119X higher for the NA rates in comparison to the reference, and almost 2X higher for the MN and 8X higher for the NA rates, in winter and summer, respectively. One specimen of the São Vicente sample presented a tumor in the caudal fin.

For both collection sites, there were no significant differences between the responses observed in summer and in winter. However, in the animals from the Cananéia estuary, the NA values were slightly higher in summer than in winter.

There were no significant differences related to sex, gonadal maturation and the biometric parameters in the occurrence of MN and NA in either sites or seasons.

Cytogenotoxicity analyses have been considered an efficient pollution indicator, especially in view of their sensitivity and correlation to the environmental contamination. Also, genotoxicity data can be used as early warning signs of degradation, allowing the implementation of control measures whenever a biological risk is detected (Pisoni *et al.*, 2004).

Higher MN and NA rates were found in fishes from the São Vicente estuary and can be understood as an effect of the pollutant inputs from irregular industrial landfills, domestic sewage and industrial effluents in this area, where metals, detergents, petroleum hydrocarbons and chlorinated hydrocarbons are present at levels considered toxic to the biota (Cesar *et al.*, 2006; Sousa *et al.*, 2007; Abessa *et al.*, 2008; and 2001 water quality report for the São Vicente estuarine system). Similar results were found in India by Mallick and Khuda-Bucksh (2003) in Centropomidae specimens from an estuary contaminated with PAH and PCB.

Although the MN rates presented higher values in fishes from São Vicente collected in summer, no significant seasonal difference was observed. The increase in NA frequency found in the organisms from Cananéia in summer was considered as a natural variation and was similar to the values found by Mallick and Khuda-Bukhsh (2003) for Centropomidae from a non-polluted tropical estuary.

The presence of a tumor in a specimen from São Vicente may be considered as an effect of contaminants with a carcinogenic, mutagenic and teratogenic potential. The water 2001 quality report for the São Vicente estuarine system (see Internet resources) observed the bioaccumulation of naphthalene (220 μg.kg^-1^) and other PAHs and PCBs in snooks from the Santos Estuarine System. Also reported were copper and mercury concentrations above the levels established in Brazil for human consumption (Cu: 35.50-39.60 μg.g^-1^ and Hg: 0.69-~5.0 μg.g^-1^) in crab and mussel tissues, and high zinc concentrations in snook, mullet and crab tissues (54.8, 63.5, and 54.8-61.5 μg.g^-1^, respectively). Dibenzo(a)anthracene was found in oysters at levels slightly below the limit for human consumption established by the U.S. Environmental Protection Agency (USEPA) (104 μg.kg^-1^), and PCBs above the limits established by USEPA in fish, mollusc and arthropod tissues (45.05, 55.40 and 27.13, respectively). Aquatic organisms with dioxins and furans in their tissues were also found.

It can be concluded that the environmental quality (especially the chemical contamination of sediments and small organisms which are predated by *C. parallelus*) is probably inducing the genetic responses detected in *Centropomus parallelus* specimens from the São Vicente estuary. In a broad view, these cytogenotoxic effects may ultimately induce physiological damage and increasing levels of mutation and neoplasia in this species, leading to an ecological imbalance. Furthermore, the MN and NA frequencies of this species can be used as biomarkers of exposure to and effect of contaminants with a genotoxic potential in environmental monitoring studies of estuarine and marine ecosystems.

**Figure 1:**
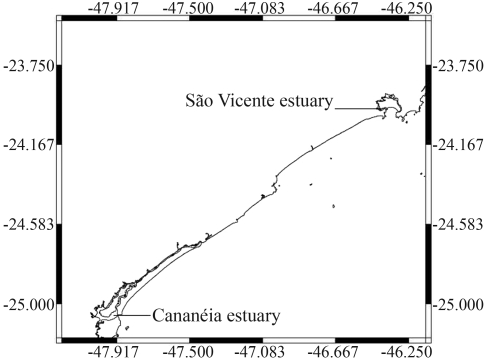
São Paulo coast showing the two areas studied: São Vicente and Cananéia estuaries.

**Figure 2:**
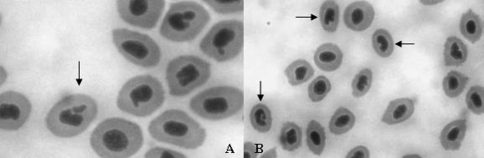
(A) Erythrocytes with micronuclei and (B) nuclear abnormalities.

## Figures and Tables

**Table 1:** Biometric parameters, sex and gonadal maturation at both collection sites in each season.

Season	Site	Specimen n.	Total length (mm)	Weight (g)	Sex	Gonadal maturation
		1	204	74.5	M	A
		2	192	55.6	F	A
		3	214	79.9	F	B
		4	216	79.8	M	B
	São Vicente	5	210	79.9	F	B
		6	183	54.5	NI	A
		7	229	106.4	F	B
		8	186	51.7	F	B
		9	183	51.1	M	A
Winter		10	247	137.5	F	B
	
		1	75	16.8	F	A
		2	88	18.2	F	B
		3	66	16.5	M	A
		4	184	22.7	F	A
	Cananéia	5	98	18.3	M	A
		6	118	19.6	F	A
		7	96	17.9	M	A
		8	72	16.8	F	A
		9	101	17.4	M	A
		10	160	21.0	F	B

		1	188	52.5	M	B
		2	183	51.0	M	B
		3	194	56.4	F	B
		4	193	66.8	F	B
	São Vicente	5	222	84.2	F	B
		6	216	98.1	M	B
		7	221	111.2	M	C
		8	212	79.6	F	B
		9	178	47.4	F	B
Summer		10	171	42.0	F	B
	
		1	90	17.8	F	A
		2	145	21.0	F	A
		3	86	17.5	M	A
		4	311	28.0	F	A
	Cananéia	5	141	21.3	M	B
		6	174	23.0	F	A
		7	171	22.5	F	A
		8	104	18.5	F	A
		9	72	16.2	M	A
		10	125	20.7	F	A

**Table 2:** Incidence of MN and NA in *C. paralellus* from São Vicente and Cananéia estuaries.

Season	Site	Cells with MN (%)^a^	Cells with NA (%)^a^
Winter	São Vicente	0.325*	3.575*
	Cananéia	0.0325	0.03

Summer	São Vicente	0.125	2.935*
	Cananéia	0.065	0.355

* Significant difference in relation to Cananéia estuary in the respective season for p < 0.05. ^a^ Frequencies in 1000 cells.
